# Clinical ethics dilemmas in a low-income setting - a national survey among physicians in Ethiopia

**DOI:** 10.1186/s12910-019-0402-x

**Published:** 2019-09-13

**Authors:** Ingrid Miljeteig, Frehiwot Defaye, Dawit Desalegn, Marion Danis

**Affiliations:** 1 Bergen Center for Ethics and Priority Setting, Department of Global Public Health and Primary Care University of Bergen, Bergen, Norway; 2 Department of Research and Development, Helse Bergen Health Trust, Bergen, Norway; 3 Addis Center of Ethics and Priority Setting, College of Health Sciences, Addis Ababa University, Addis Ababa, Ethiopia; 4 Department of Bioethics, National Institutes of Health, Bethesda, USA

**Keywords:** Ethics, Clinical ethics, Low-income country, Ethiopia, Health worker, Dilemma, Physicians

## Abstract

**Background:**

Ethical dilemmas are part of medicine, but the type of challenges, the frequency of their occurrence and the nuances in the difficulties have not been systematically studied in low-income settings. The objective of this paper was to map out the ethical dilemmas from the perspective of Ethiopian physicians working in public hospitals.

**Method:**

A national survey of physicians from 49 public hospitals using stratified, multi-stage sampling was conducted in six of the 11 regions in Ethiopia. Descriptive statistics were used and the responses to the open-ended question “If you have experienced any ethical dilemma, can you please describe a dilemma you have encountered in your own words?” were analyzed using a template analysis process.

**Results:**

A total of 587 physicians responded (response rate 91,7%), and 565 met the inclusion criteria. Twelve of 24 specified ethically challenging situations were reported to be experienced often or sometimes by more than 50% of the physicians. The most frequently reported challenge concerned resource distribution: 93% agreed that they often or sometimes had to make difficult choices due to resource limitation, and 83% often or sometimes encountered difficulties because patients were unable to pay for the preferred course of treatment. Other frequently reported difficulties were doubts about doing good or harming the patient, relating to conflicting views, concern for family welfare, disclosure issues and caring for patients not able to consent. Few reported dilemmas related to end-of-life issues. The 200 responses to the open-ended question mirrored the quantitative results.

**Discussion:**

Ethiopian physicians report ethical challenges related more to bedside rationing and fairness concerns than futility discussions and conflicts about autonomy as described in studies from high-income countries. In addition to the high report of experienced challenges, gravity of the dilemmas that are present in their narratives are striking. Recognition of the everyday experiences of physicians in low-income settings should prompt the development of ethics teaching and support mechanisms, discussion of ethical guidelines as well as increase our focus on how to improve the grave resource scarcity they describe.

**Electronic supplementary material:**

The online version of this article (10.1186/s12910-019-0402-x) contains supplementary material, which is available to authorized users.

## Background

All health workers are confronted with ethical challenges in the course of their clinical practice. Numerous empirical studies describe health workers’ dilemmas and decision-making processes [1–4]. But, these studies focus on high-income countries –few studies present or discuss findings from low- and middle-income countries (LMIC), and the available studies are mostly small qualitative studies [5–10]. Yet there are many features of health care delivery in low- and middle-income countries that would lead to the expectation that health workers in these countries experience many ethical difficult situations; they are confronted with high patient-rosters, few resources, limited and relatively newly implemented advanced medical diagnostics and treatments like MRI machines, ventilators and cancer treatments as well as diverse cultural, religious and socio-economic contexts. But what ethical challenges are they experiencing most frequently and how do they describe the most challenging dilemmas in their own words? Our objective is to explore these questions and map out the ethical dilemmas from the perspective of Ethiopian physicians. Dilemmas are here understood in a broad way, reflecting how clinicians often use the term, which is broader than what philosophically would be described as an ethical dilemma. Clinicians often use the word “dilemma” to describe ethical challenging situations where there might be an obvious ethical solution, but it might be hard to reach due to various barriers. They also sometimes use it to describe other clinical situations which challenge their values. This understanding of dilemma is also used in other studies [2].

Here we present both quantitative and qualitative data from an extensive, representative survey of physicians in Ethiopia, the second most populated country in Africa. While Ethiopia is still far behind on human development indices, considerable efforts have been made to improve health conditions among the population [11–13]. Increasing the number of qualified health workers is one of the strategies [14]. Also, while the overall priority has been to improve essential public health services and primary care, more individualized preventive care to reduce cardiovascular disease and more technologically advanced care such as dialysis, and cancer therapy are available in selected institutions. Private, out-of-pocket expenditures are high. Similar to many other African countries, the majority of the population lives in rural areas, and still partakes of traditional medicines and reflects great religious diversity.

In this first effort to describe clinical ethical dilemmas in Ethiopia, we focus on physicians working in local, regional and referral hospitals in both rural, pastural and urban areas, including both specialists, general practitioners (GPs) and residents with more than 1 year of practice after internship.

## Method

### Study population

A national survey was conducted among physicians from 49 public hospitals using stratified, multi-stage sampling in six of the 11 regions in Ethiopia. That included local hospitals with only few physicians, regional hospitals and large referral hospitals. To obtain a representative sample of categories of regions (urban, rural and pastoralist), we randomly selected two regions from each category (six of 11 regions). The region of Addis Ababa was purposively included as most specialized physicians work in the capital and we wanted to make sure to get their responses. All physicians in the selected hospitals were invited to respond to a self-administered questionnaire. The data collection was done from July to November 2013. In a previously published paper presenting data from the same survey, extensive descriptions of the survey methods can be found [15].

### Survey instrument

In this paper, we present results mainly from two questions. The first question asked how often in the two last years they had encountered ethically challenging situations. To answer this question, we asked them to check a list of predefined situations. The development of this question had a sequential exploratory, mixed method design [16]. That is, we first collected qualitative data in order to develop a new quantitative instrument for exploring the phenomenon. This was done in an ethics-training program of future teachers in medical ethics in Ethiopia in which 25 Ethiopian medical academic doctors participated over 3 years [17]. They specialized in various disciplines and had lengthy experience working in various Ethiopian hospitals. Through the 3-year program, multiple cases were shared and discussed. The cases were systematized in themes and emerging dilemmas and nuances of the dilemmas previously used in a similar European study were gathered [2]. The list of situations was then further developed with input from the participants and was pilot-tested among a sample of 20 physicians at various institutions in Ethiopia. The final list of situations included nine categories of 24 ethical challenges. Some of the situations listed are more general, while others are more specific. Response options related to the frequency of encountering a situation: often/sometimes/rarely/never or not applicable (The questionaire is available in Additional file 1).

The other question from the survey we used in this paper was an open-ended question: “If you have experienced any ethical dilemma, can you please describe a dilemma you have encountered in your own words?” The respondents could respond to this question in English or Amharic.

### Analysis

#### Statistical analyses

Data were coded, entered using EPI INFO, cleaned, and were analyzed using Stata13.1 statistical software. Responses were analyzed using descriptive statistics.

### Qualitative analysis

Responses to the open-ended question were analyzed using a template analysis approach [18–20]. In this approach, predefined categories are used when analyzing the text. We used the predefined categories from the initial qualitative material when coding phrases in the text. We chose this method because it is suitable for the development of new descriptions and because we wanted to use the same categories as we had done in the quantitative question, providing narratives to gain in-depth information regarding the dilemma. The full coding was done manually by one of the authors (IM), while (FBD) and (MD) coded parts of the material.

## Results

### The respondents

Of the 640 practitioners to whom questionnaires were distributed, 587 responded (response rate 91,7%). Questionnaires from physicians with less than 1-year of service were excluded and final analysis was done on data from 565 respondents. Most respondents were male (78%) and young (mean age was 31.1, median age 28 years), and had less than 6 years of medical practice (ranging from 1 to 32 years) (Table 1). Half of them were general practitioners, while approximately ¼ were specialists and ¼ residents. Many had long working hours (average 46 h in government hospitals), saw many patients during a week, and 38% also worked in private clinics.

**Table 1:** Respondents Characteristics. All respondents were government employed. Analysis was done on valid N, excluding missing and not applicable

		Number who answered this question
Women/Men (%)	21 /79	563
Mean Age (Range)	31	555
Age group (%)		
< 31	68	555
31–40	21	
41–50	9	
> 50	4	
Undergraduate medical training Ethiopia (%)	94	551
Postgraduate medical training Ethiopia (%)	94	278
Mean service year	6	540
Years in practice (%)		
1–5 years	70	540
6–10 years	15	
11–20 years	9	
> = 21 years	8	
Professional status (%)		
GPs	49	556
Specialists	24	
Residents	27	
Have private practice	38	565
Average work hour/week in government	46 (SD = 29)	525
Average work hour/week in private	20 (SD = 11)	28
Average number of patients/week	135 (10–600) (SD = 92)	525
Involvement in medical academics (%)	72	
Involved as:		
Instructor	53	413
Resident	36	
Researcher	6	
Others	6	
Involvement in planning and decision-making at the hospital (%)	28	559

### Dilemmas experienced

**Fig. 1:**
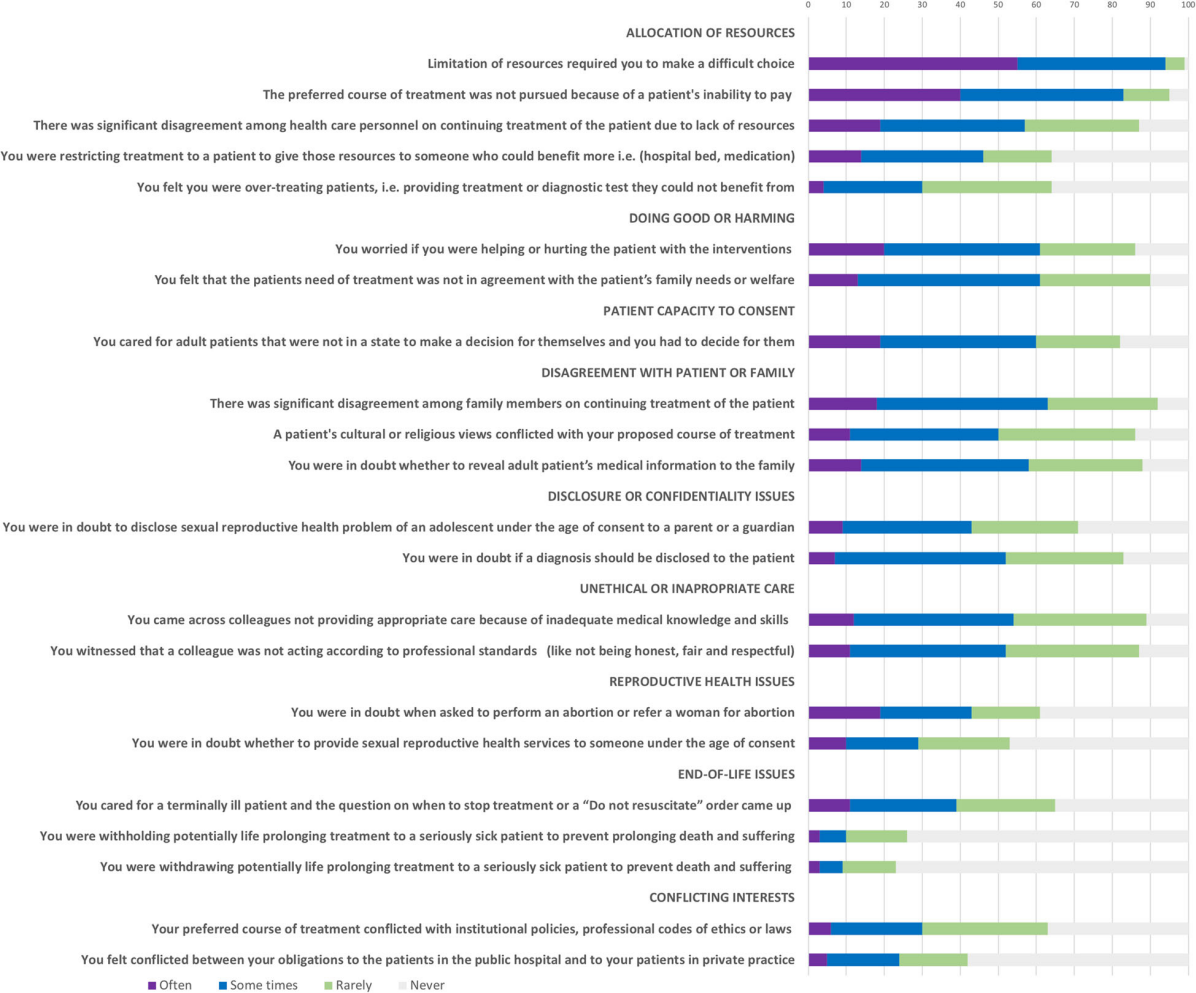
Physicians’ reported dilemmas

The 24 presented ethical challenging situations were sorted according to how often the physicians reported to experience them, and are grouped in nine themes (see Fig. 1).

Twelve of the specified challenging situations were experienced often or sometimes by more than 50% of the physicians. The most frequently encountered challenges concerned the allocation of resources; 93% agreed that they often or sometimes had to make difficult choices due to resource limitation, and 83% often or sometimes encountered dilemmas because patients were unable to pay for the preferred course of treatment. Difficulties concerning doubts about helping or hurting the patient with the intervention, conflicting views in the family or concerns for effects on the family welfare, caring for patients not able to consent or disclosure issues were all frequently experienced. Also, situations involving observation of colleagues who were not providing appropriate care due to lack of knowledge or skills or acting unethically towards patients were commonly encountered. Dilemmas concerning withholding or withdrawal of life-prolonging treatment of severely sick or dying patients and requests of euthanasia or assisted suicide were reported to occur often or sometimes among less than 10% of the physicians.

In six of the nine categories, some of the listed ethical challenging situations were reported to be often or sometimes experienced among more than 40% of the respondents. Only reproductive health dilemmas, end-of-life dilemmas, and conflict of interest dilemmas were reported less than among 40% of the respondents.

### Results from the qualitative analysis

Of the 565 respondents, 200 responded to the open-ended question “If you have experienced any ethical dilemma, can you please describe a dilemma you have encountered in your own words?” Most of them provided one or several examples described in details, while others presented bullet-point lists of dilemmas they had experienced themselves or situations they found ethically challenging in general. Examples of all the nine categories of dilemmas we had included in the survey were presented. Dilemmas that our predefined categories did not capture were dilemmas arising as a result of the lack of laws or guidelines regulating the situation or the lack of training, tools or experts aiding the physician in how to handle a challenging situation.

As in the quantitative question, the most frequent examples were ethical challenging situation concerning resource allocation and the families’ economic situation, as one physician wrote in bold letters “*Limitation of resources is the major challenge*” . Other frequently reported stories concerned disclosure of information and unethical behavior among colleagues; quite frequent were dilemmas concerning abortion and conflicts arising due to cultural/religious aspects while fewer described dilemmas relating to doubts about withholding/withdrawal of life-prolonging treatment or conflicting interests. No examples concerned a request for assisted suicide or euthanasia and very few concerned making decisions for patients unable to consent. While relative few of the respondents reported to experience reproductive health challenges, 21 of the 200 who responded to the question mentioned abortion as an ethical dilemma or described in detail a concrete dilemma related to abortion that they had experienced.

As the narratives coded under the category “Doing good or not harming” and some of the narratives that belonged to the category of “Conflicting interests” were overlapping, we combined these in a new category we called “Ambiguity about beneficence and conflicting interests.” Quotes from the category “Patient capacity to consent” fitted better in the category “Disclosure and confidentially issues.” Below we present the revised seven categories in detail with quotes.

### Dilemmas concerning resource scarcity and allocation of resources

*I am not going to mention any specific situation because my everyday clinical experience is filled with cases where I have to go into a dilemma concerning the allocation of the resource with the patient's need and requirement. It is very stressful subject for a clinician and the patient his/her family. I think this issue is presented at all health care provider levels in rural and urban areas and needs all of the attention it can be given.*

Numerous specific situations regarding priority and scarcity were presented. Also, many of the other difficulties that were reported occurred as a result of lacking resources. For example, a dilemma regarding whether to provide an abortion or not happened in the setting where the woman begged for this as she could not afford to go to the private clinic.

Below we present two major themes under the category of resource scarcity and allocation: being responsible for providing suboptimal care or no care at all, and the distribution dilemmas.

### Responsible for providing suboptimal care or no care at all

A considerable proportion of the described dilemmas concerned situations where diagnostic options, medicines, hospital beds, surgical capacity or referral opportunities were not sufficiently available, and physicians had to compromise the quality of their service in one-way or another. Limited staff and lack of qualified staff also led to suboptimal treatment which was reported as ethically challenging. Many physicians were frustrated by having to provide less efficient care; care that did not meet recommended guidelines or comport with teaching in medical school, or treatment they considered being potentially harmful to patients.

*I am an orthopedic surgeon, and there is a big demand for our service in our hospital. But because of limited resource available, we generally use non-surgical methods that are time-consuming for the patients and lead to major complications and disability. The high bed occupancy rate and the less satisfaction of the patient are why I am always uncomfortable regarding my job.*

Physicians reported prescribing too short a course of medications due to unreliable stocks of medicines, treating patients as outpatients instead of admitting them to the ward when no beds were available or letting patients stay in the local hospital because the distance to the referral hospital was too far. Also, the lack of capacity to perform laboratory tests led to suboptimal treatment, as one described:

*“I am sorry to say that I work in a referral hospital without blood bank, electrolyte machine, etc. The situation forces you to do non-ethical things. I know I have given KCL (potassium chloride) to a diabetic patient who had ketoacidosis without checking the K (potassium) level in the blood. That can be fatal.”*

Three respondents described their ethical concerns with providing overly broad-spectrum antibiotics because their healthcare facility lacked the capacity to determine the antibiotic sensitivity of an organism when treating an infection.

### Distributive dilemma

Several respondents wrote about situations in which patients had competing needs in the face of insufficient resources for treating both. One informant described how this is a daily part of his job in the obstetric ward.

*It is my daily duty hours experience to deny patients (laboring mothers) resources of care, especially admission beds, in order to prioritize one over another based on their situation (diagnosis). Since resources are never enough to accommodate every laboring mother.*

Several respondents explained how the strategy of” first-come, first-served” was typically used and how they disagreed with it.

*I am not able to give priorities to those who benefit more, only because there are many patients who are registered before them (even if they get little benefit).*

*Mothers should be prioritized due to their clinical condition; like mothers who have cervical cancer stage IIa which can be operated. But they are kept on the waiting list of the hospital chart like any other patient to be admitted. When they get the chance/bed in the hospital, mostly the disease stage is advanced and not possible for surgical intervention. They are transferred for radiotherapy. They wait additional 4-6 months, and at the end, they (most of them) end up with death.*

Others explained how they aimed to prioritize the sickest or the one who could benefit the most first. One respondent provided insight into the challenging decision-making when resources were lacking.

*Usually, in my duty I face situations due to lack of resources: like bed, OR (operation room) materials, sometimes O*_*2*_ *(oxygen) and other resuscitation materials. It makes me choose the most critical patient who is going to suffer immediate complication to give priority. But at the end those who have been labeled low risk later come with more complication than those who were given the priority. And I feel bad for being poor, working in resource-limited setup. Whatever you wanted to do is limited on what is available at hand.*

### Ambiguity about beneficence and conflicting interests

Many respondents described the challenges of treating poor patients in a setting with high out-of-pocket expenditures. They referred to their role and responsibility in these situations. One said: “*We are risking to disrupt families for the sake of the patient”* In many instances, physicians knew that their decision would have consequences for the family economy as well as the patient’s health.

*"The dilemma is; patient versus the family."*

The anguish of what to do became particularly strong if they saw or knew that the family would waste their money or go into debt.

*I once had an experience that a child had ARF (Acute Renal Failure) requiring dialysis. I consulted the parents about the option. I knew that the child might require dialysis lifelong and the parents were from the countryside and had a large family whom they needed to support. Selling their property to pay for the dialysis fee would be required to do so. I felt like I was disrupting their life for something that has little hope.*

Several wrote about situations where the patient or the family could not pay, and they themselves paid.

*I regularly encounter patients in the emergency department who need admission and require medication for life-threatening diseases; however, some of them are economically challenged and cannot afford even 5birr/day, for a hospital bed, let alone the third-generation medication that will be administered for a week or longer. So, it is very sad and heartbreaking as physician coming across such patient and basically watch them die. Often my colleagues and I empty our wallets so they could get admitted and start on the treatment.*

### Dilemmas concerning disclosure, confidentiality and patient’s capacity to consent

A substantial number of dilemmas concerned confidentiality and disclosure of relevant information. Physicians reported that these dilemmas were particularly challenging if the pa

tient was not able to consent, was a minor, or had less power within the family (being a woman).

*(…*) *She said:* “*I rather die than my parents to hear this!” (in a case describing a woman below 18 years requesting abortion)* Situations in which patients requested non-disclosure of information to others most often concerned a patient’s HIV infection or a patient’s request for or receipt of an abortion. Some of the HIV-cases reveal the concern for individuals other than the patient and the distress of knowing without intervening.

*A month back a newly diagnosed HIV stage IV-patient was admitted. The next day, his family refused to help or buy any drug unless they were informed about his HIV status. By then the best thing I found was to tell them another chronic illness that can be transmittable via blood/fluid borne illness. They were convinced. What would you have done???*

Another dilemma concerned the requests to the physicians from families to not tell the patient suffering from a severe disease (like cancer) the name of the condition or the prognosis.

( … ) *I knew she had every right to know what was happening inside her; months she had to live. But her husband didn’t allow me, so I had to put on a brave face and tell her everything was going to be okay!!*

While the dilemmas above regarding disclosure concern situations when others asked physicians not to disclose, the third type of disclosure dilemma concerns the physician’s own doubts about disclosure of information to the patient to protect them from fear or family conflicts.

*Sometimes I get reluctant to tell patients or their relatives on the prognosis of the treatment (operation on my case) because they are mostly illiterate and naturally afraid of operations. The patient may refuse and has not got the chance for treatment even though being counseled on every detail of the treatment.*

Respondents described decision-making traditions sometimes leading to delay in treatment because the patient could not decide to start treatment and pay for it until older family member had to come to the hospital. *S*everal physicians referred to episodes where they feared that the family might abandon the patient if they did not stick to the family decision.

### Dilemmas concerning cultural issues and disagreement with or within families

A number of cases described challenges of treating and communicating with patients and families in a diverse cultural context. Several of the cases concern situations where, as one respondent describes it: “*Patient’s cultural or religious view often conflict with the proposed course of treatment*.” These situations often involved diverging understanding of disease and necessity of medical treatment or diagnosis such as getting acceptance from Muslim family members to perform a pelvic examination on delivering mothers, not accepting medical advice because of taboo or traditional healers’ provision of contrary advice. Many described how they tried to respect the patient/family view despite opposing it.

*The family members of the patients prefer to go to holy water to get recovery from their mental illness. In such cases, I tried to respect their religion and to accept their preference, but also tell them that medications also should be taken at the same time to get a better outcome.*

Others found this challenging, also because it sometimes led to increased personal obligations for the physician.

*I witnessed the difficulty of donating blood; even when it’s their close relative in severe anemia. They think it is taboo. For bleeding mothers, we physicians were obliged to give (donate) for them. This is a big challenge I witnessed in society.*

In several instances, physicians described their reactions and responses to the challenge posed by conflicts they encountered with families. Physicians described that it was particularly hard to see families taking home their children from the hospital against medical advice.

*A father took his child home against medical advice for surgical intervention. It was a four years old child with intestinal obstruction. I still feel guilty for not intervening.*

### Dilemmas concerning reproductive health issues

The most significant number of reproductive dilemmas concerned physician’s doubts and challenges related to the provision of abortion. But some also described dilemmas with female genital mutilation. One physician wrote: “*One dilemma occurs when a woman with FGM (female genital mutilation- 4*^*th*^ *stage) come to give birth and we have to ask her husband (to give permission) to make an incision of the external part of the vagina”.* In the abortion cases, respondents questioned whether they were doing right, and some made references to the sinful act of killing or wrote about tragic circumstances of young women dying. They described their regrets and as well as their obligations to prohibit harm.

*I encountered a lady who wanted abortion to be done. I had to refuse since the hospital did not allow unless there are indications. She had an abortion outside (ie unsafe). After ten days she came back with severe sepsis and died in the hospital. I felt I could have done safe abortion, which could have prevented sepsis, and I felt guilty. Later a similar patient appeared apneic. I admitted her with a false diagnosis and did abortion, fearing the same thing might happen.*

The abortion law in itself was leading to problems for some of our responders, as abortion is only allowed under specific conditions, which many women do not fulfill. Aiming to reduce one of the world’s highest maternal mortality rates, Ethiopia slightly liberalized their abortion law in 2005 from abortion being absolute illegal to allowing for abortion if the woman fills specific criteria: incest, rape, minor (under 18), fatal fetal condition or if not fit physically or mentally to be mother (needs to be assessed by trained health worker) [21]. In our material we had several versions of dilemmas experienced when the woman did not fulfill the criteria and the provider still felt abortion to be indicated.

*In case of abortion where it may not be possible to perform an abortion by the country's law, but you may feel that she should be helped even though it is against the code of the country.*

A unique condition in the law is that the law specifies that for rape and incest the word of the woman pertaining to the offense is enough and no further investigation or evidence is needed [21]. Some of our informants described how this forced them sometimes to accept her claim even if they found it unlikely to be true. One wrote: “*I have encountered patients who came claim to be raped and got pregnant even though the real story is different.”* While several physicians explain how they cannot perform the abortion themselves due to religious conviction, some of them also made clear that they tell the woman where to go to get it done.

In several of the abortion cases, physicians wrote of their religiosity or perceptions of ethically right actions. One presented a strong statement against abortion and his/her opinion that *“government forcing medical professionals to do actions which contradict against humanity!”*

### Dilemmas concerning end-of-life issues

Few of the cases presented were about end-of-life issues like withholding or withdrawal of life-prolonging treatment or concerns about “going too far.” Physicians working in larger referral hospitals reported all the described cases regarding end-of -life decisions. One respondent explained how new equipment is leading to previously unknown dilemmas, describing situations where they were keeping brain-dead patients on the mechanical ventilator because stopping the ventilator is culturally difficult and there are no guidelines for how to withdraw treatment. In the cases presented, the challenges of proper diagnostics, legal regulations, and their own roles were described. The concern for efficient use of resources was also significant.

*I had few patients who were critically ill being managed in the ICU; one patient clinically brain-dead on mechanical ventilator, another patient persistent vegetative state. Both of them were of very poor prognosis. I had no legal ground to decide on the termination of treatment, and even in confirmation of the diagnosis as we have a lack of resources like arterial blood gas analysis, EEG and such alike. They stayed in ICU for a long time, and they occupied a very scarce resource; the mechanical ventilation, which was actually very ambiguous and controversial issue for us.*

No physicians described circumstances in which they had been asked for euthanasia or assisted suicide. But some referred to the challenges of treating severely sick patients and the fear of being accused of killing the patient if they stopped life-prolonging treatment.

*Even as a health professional we know the patient is in end state (going to pass) we do not stop treatment and treat him until he passed away. We do not negotiate with the family to terminate the medication due to cultural issues.*

### Dilemmas concerning observed unethical or inappropriate care

There were various examples of unethical or improper care. Some physicians reflected on situations in which they had behaved unethically; providing care they were not skilled enough to do or did not know how to do because there was no one else who had more advanced training or they lacked practice guidelines.

*Often you will be forced to choose one of the two evils: You intervene beyond your specialty out of desperation up on family/patient's consent, because they cannot afford to go anywhere else. Like gynecologists urged to do surgical cases or internists pushed to handle surgical cases. (The scenario is when that best person or specialist is not available).*

Others were cases where they had observed colleagues or staff with poor attitudes or behavior. Several cases concerned private clinics. The majority of cases related either the use of unnecessary testing or treatment in private clinics and the unethical requests from poor patients to pay for this, or colleagues referring patients to their private clinics or bringing their private patients into the public hospital to use the equipment there.

Other described how their enormous workload or the structural environment led to dilemmas.

*( … ) In government health facilities the number of doctors is not proportional to the number of patients visiting. So many physicians will be in dilemma whether to see or not the critical patient who comes last, after doctors are exhausted. In short, if a doctor cannot see maybe the 81st patient, he will be accused and punished. No one appreciates what he did.*

The cases describing unethical behavior were described more emotionally than many of the other narratives. Some dilemmas pertained to the hierarchy within their hospital/unit and the absence or disregard for laws or regulations. Physicians saw no means to enforce ethical behavior or complain if something unethical was done.

*… and there is no one to tell about something unethical that somebody has done. So you just pray that tomorrow would be better than today and move on.*

## Discussion

Ethiopian physicians encounter a great variety of ethical challenges and by inviting them to tell about their dilemmas, we have a detailed and nuanced picture of what is at stake. In addition to the high frequency of experienced challenges, the gravity of the dilemma that are presented in the description of these dilemmas is striking. The difficulty of handling resource scarcity and the consequences of such scarcity are remarkable and warrant further attention. The other types of cases are not entirely different from the profile of dilemmas that are reported in high-income countries, but the circumstances in this low-income country make the reactions and responses of physicians and those around them somewhat different. The lack of guidelines and regulations provide less support and encouragement of ethically sound behavior, and the lack of ethics training leaves physicians unprepared to respond well. Below we will discuss our findings in more details.

### Bedside rationing and fairness concerns rather than futility discussions and conflicts about autonomy

Both the ranking of ethical challenging situations and the response to the open-ended question show that respondents often encountered dilemmas related to resource scarcity, bedside rationing and fairness. This might not be surprising if we consider the working conditions in government hospitals in a low-income country. In interpreting this finding, we found no other data from other low-income countries for comparison. There are however some data from high-income countries. Saarni et al. studied all specialists in Finland from 2007 and found that 20% of the participants experienced ethically questionable treatment decisions due to resource scarcity; at the same time, except in psychiatry, all specialists found that overtreatment was a much more frequent dilemma than undertreatment and patient rights issues [22]. In Hurst’s study among general practitioners and internists, approximately 50% of the participating physicians reported that they had experienced ethical dilemmas due to scarcity of resources [14]. Dilemmas due to scarcity of resources were ranked as the 9th most common among their 13 listed dilemmas. Hurst’s study was conducted in Switzerland, Italy, UK and Norway. The health expenditures per capita in current USD was 7477 in Norway, 2738 in Italy, 9835 in Switzerland and 3958 in UK in 2016, while in was 27,52 in Ethiopia [23]. The gross difference on available resources makes the reality our respondents work in very different, and this is reflected in the ethical challenges they face.

Few among our responders reported experiencing dilemmas due to concerns for prolonging or starting life-prolonging treatment of severely sick and dying patients, requests for euthanasia or assisted suicide. This is different from other studies [14]. Explanations might be that very few hospitals have equipment like ventilators, dialysis machines, etc., and also as several respondents pointed out, there is no tradition for this kind of decision making in the Ethiopian culture. Our experience as physicians and teachers in Ethiopia confirms this finding. Withdrawal of life-prolonging treatment hardly happen, except if it is on the family request or if the family cannot pay any longer, or in some cases, if they need the equipment for someone else. The most common reason why families ask to withdraw is that they want to take the patient home when still alive. Transporting a dead body is very expensive. With a growing elderly population, rising incident of non-communicable diseases (NCDs) in the society and increased survival of people with disability, as well as the implementation of health care packages including cancer-treatment, more surgery and intensive care, the frequency of these dilemmas is likely to increase. Our study points at the need for developing proper procedures and guidelines to support these types of decision-making among physicians and families.

### Perceived responsibility and handling of non-medical issues in patient care

Our Ethiopian respondents expressed substantial concern regarding non-medical issues like protecting family financial welfare or avoiding discharge due to cultural issues, which is showed both in the quantitative and qualitative part of the survey. Also, some physicians gave examples of how they pay personally, and even donate blood to the worst-off patients. We interpret this whole picture as an illustration of their strong commitment and sense of obligation to do what is best for their patients, and the personal burden that follows. Both the likelihood of physicians providing charity care and donating blood has been reported previously [24–26], but we could not find any other studies describing physicians’ narratives of paying for their patients’ medicines or rapidly donating blood to avoid their patients of dying in front of them. This finding should inspire further research on how health personnel navigate in resource-constrained settings and get personally involved in the treatment of their patients.

In the cases where they write about conflicting interests between patients and family welfare, their concerns for non-medical issues become explicit. In other papers from the same survey, we find that Ethiopian physicians report a strong sense of obligation to protect against catastrophic health expenditures as well as a significantly higher priority for protecting family finances rather than protecting the institution from high costs [27]. They report putting substantial weight on the ability to work and the patient’s role as sole economic provider for the family in cases where they have to decide whether or not to give priority to a patient in need of expensive treatment [28]. The narratives provided by our respondents offer illustrations of how problematic the physicians find their perceived extended responsibility when they know the catastrophic financial consequences of a recommendation or request for a family in severe economic distress. While our findings point at the responsiveness of some physicians, they also highlight the need for more widespread moral deliberation about how to resolve the competing value of individual health versus family welfare.

### The high moral burden of not providing what the patient needs

The majority of the cases described in our study concern the experienced dilemmas and underlying frustration of not delivering what seems to be in many cases very basic, and not exceptionally costly, treatment to the patient due to either lack of available treatment options or the family’s inability to pay. The majority report and describe this to happen often. In previously published papers from the same dataset, we present data showing that 29, 23 and 25% reported that they daily, weekly or monthly experienced situations where a patient suffered adverse consequences as a result of limited resources in the health care system. Among the consequences they have seen, 54% had encountered deaths, 19% acute life-threatening events, while 15% had encountered permanent or temporary disabilities that they attributed to scarcity of resources. Also, 36% on a daily and 23% on a weekly basis reported that they had been so troubled by limited resourses that they regreted their profession [15]. Given the high volume of patients they see and their report of dilemmas related not providing what the patients need indicates that they experience a high moral burden of handling these cases. We did not ask specifically about their experiences of moral distress or stress symptoms, and can therefore not report on that. Though, our experiences from the field as physicians and teachers confirms what we find as the underlying vibe in our results – a great distress of not being the doctor you want to be, given the challenges you have to deal with. This should be an area of prioritized research, as more knowledge on health providers moral burden and moral distress is noeeded in order for lecturers, leaders and policymakers aiming at ensuring ethical sound decision making and decisions and at reducing conflicts and burnouts [29].

### Adjustment of standards and negotiating in conflicts

Our participants reported that they struggled with accepting ethical opinions and decisions that diverged from their own, and many of them explained how they either had to adjust their own standards or convictions or act in ways that were contrary to what they considered to be right. This was most clearly seen in the narratives about how they agreed to lie or not tell the patient about their condition to protect the patient or the family, or how they handled situations when women asked for an abortion. In some of the descriptions about challenges related to abortion, the respondents report how their religious and personal convictions led them to believe that the act was ethically wrong, their concern for the women’s lives and the commitment to reducing maternal death made them do it. Also, other studies from countries where the abortion law is only slightly liberalized show how many providers are deeply against abortion, but still provide it to avoid greater harm [30]. Studies among these providers have shown a considerable proportion of moral distress [10, 31]. In our material we also found that some resisted to perform abortion even if it was allowed, but they found ways to help the woman getting in contact with someone who could do it.

The participants were challenged in handling patients and making decisions when the patient or the family had a different cultural, religious or socio-economical background. In the quotes where the physicians write about how they are asked to allow use of holy water, herbs or taking the patient to priests, they describe how they struggle between respecting the patient or family, but at the same time ensuring what is best for the patient. While many of them explained how they tried to adjust to what the family wanted or negotiate to a certain extent to prohibit harm, several also referred to their impotence in decisions concerning influence on the final outcome. In a country without a proper welfare state that can take care of children or others in case the family cannot, and without protection laws for children and other vulnerable populations, the providers have fewer options to go against the family will.

### Professionalism, lack of standards and support

Witnessing unethical behavior among colleagues or self-reflection on personally performing unethical behavior was frequently reported. In the quotes, several questioned the ethical standards among their colleagues and in Ethiopian health care, and many underlined that they have neither training in how to handle these situations nor someone to ask for support. In a previous study, we found that they have few or no ethical guidelines [15]. They work in a setting with few health workers and an overload of patients. Time for reflection, team discussions and colleague support are minimal. Our participants saw many patients every day, and many explain how they were stretched beyond their limits both in the number of patients they were required to treat, procedures they are trained to perform or roles they are supposed to fill. Several of them asked for guidelines or questioned the legal regulations that were not entirely in line with the contemporary situation. This became particularly evident in the cases described on ventilation of brain-dead patients or in the examples where parents withdrew children from treatment that was expected to be beneficial.

The overall picture we see in our material is the grave resource scarcity that influence the physicians in numerous ways and enforce them into ethically challenging situations and dilemmas. While training, guidelines and support might prepare and strengthen them in their decision-making and professionalism, without more resources to provide essential, quality care for their patients, financial risk protection for families experiencing health issues and more trained staff, it is almost impossible to imagine change in the dilemmatic situation. Ethiopia is doing many efforts to improve the health care system, hopefully leading to better health and less poverty among the population, and better working conditions for health workers.

### Strengths and limitations

Our study provides new and valuable empirical knowledge on what goes on at the ground level in Africa’s second largest country. The difference in experienced dilemmas from studies like Hurst et al. [2] and the extent of unique stories related to socio-economic and cultural circumstances in such a large, diverse, and low-income country provide a novel contribution to understanding the practice of medicine in such a setting. While we would suggest that our results might be transferable to other low-income settings, one might also consider how relevant some aspects might be for high-income settings. In many high-income countries there is an increasing challenge of resource scarcity due to constant development of new high-cost interventions, increasingly elderly populations and restricted health-budgets. Under these circumstances hard choices about priorities must be made.

As far as we know, this is the first study of its kind, involving a national survey of a representative sample of physicians in a low-income country, asking them both to report the dilemmas they experience most frequently and to give examples of dilemmas in their own word. We find that the narratives correspond well with the quantitative results, which we interpret as indicating a high degree of internal validity of our findings.

We also find that our study can contribute to the call for more studies from low-and middle-income settings as the whole field of bioethics is heavily weighted towards detailed studies from high-income settings and that the majority of studies from low-income contexts concerns dilemmas related to informed consent and research ethics dilemma [32, 33].

Another strength of our study is the rigorous way we developed our survey instrument and the high response rate we got. But our study is not without limitations. We used predefined dilemmas and categories in the survey instrument and the analysis. The responses for how often the ethical challenging situations were experienced, were unspecific and subjective (often, rarely, never), and we cannot exactly quantify how often they experience the situations. Also, our interpretation of the open-ended question should be read with caution. Although two of us are Ethiopian physicians and we all know the Ethiopian health care system well, we might misunderstand or misinterpret what our respondents meant, and we read our expectations into the material. Also, the culture and contexts in Ethiopia are so diverse, so we cannot claim to know it completely or comprehensively. In our paper, we have therefore tried to bring as much primary data to the reader’s attention, in the form of qualitative text. Also, we recommend that more studies be done to get more in-depth knowledge about the dilemmas at the ground level and more discussion of the meaning and implications of the results. In our study we only included physicians. We did this both because we thought that physicians are often the final decision maker, but also because our resources for conducting a survey which also included nurses and health officers, were limited. Future studies should also do sub analysis to explore if factors like hospital level, urban/rural and personal characteristics influence dilemmas experienced.

## Conclusion

The Ethiopian physicians report that they have to deal with multiple ethical challenges in their clinical work. The gravity of the situations they describe and the regularity of tough dilemmas of bedside rationing and trade-offs between individual health benefit and family welfare should get further attention from an international audience as well as among policy makers, lecturers of students and clinicians themselves. Our study makes clear that capacity-building in health care delivery in Ethiopia should include ethics training and ethical guidelines as well as deliberation about these issues among physicians, the health care leaders, and the public. While our findings map out what goes on at the ground in a resource-deprived setting, we anticipate that our study discuss dilemma most health workers can recognize, and which hopefully can lead to further reflection on ethics, decision-making, resource distribution and role of guidelines and regulations.

## Additional file

Additional file 1: Questionaire (PDF 189 kb)

## Data Availability

The data used and analysed during the current study are available from the corresponding author on reasonable request.
