# Supplementary material for: Clinical ethics dilemmas in a low-income setting - a national survey among physicians in Ethiopia
Source: BMC Med Ethics. 2019 Sep 13;20:63. doi: 10.1186/s12910-019-0402-x (PMC6743170; doi:10.1186/s12910-019-0402-x)
Supplement: Supplementary file 1 — Questionaire (PDF 189 kb) [file 12910_2019_402_MOESM1_ESM.pdf]

Values at the Bedside in Ethiopia

A Survey of Physicians Regarding Ethical Dilemmas in Clinical Practice

Thank you for your participation! Your contribution will be highly valued, as getting information from physicians working "on the ground" with patients is crucial in this first study of medical ethical dilemmas in Ethiopia. The data from this survey will help us in making teaching and training in medical ethics for students and clinicians context adjusted and relevant. It will also be useful for evidence based policy and practice in medical ethics. Since there are important differences between health care levels, some questions may seem not applicable to you, but please answer as best you can based upon your situation. We value your opinions, and hope you help us with filling out the whole questionnaire.

Part I: Background

1. Gender: 1. Male 2. Female
2. Age: ________Years
3. Where did you have your medical training?
   1. Undergraduate: Country ________________Medical school __________________
   2. Post graduate: Country ________________Medical school __________________
4. Since graduating, as MD, how many years have you been practicing medicine?_____ years
5. Are you currently working as
   1. GP
   2. Specialist in (please specify)________
   3. Resident in (please specify)________
   4. Other (please specify)__________
6. Where do you practice as a doctor? (you can check more than one if applicable)?
   1. Government institution
   2. Private for profit institution
   3. Private wing in a government facility
   4. Private non profit institution
   5. Own private institution
   6. Others__________
7. On average how many hours/ week do you spend in
   1. Government institution ____ 2. Private wing_____ 3. Private institution_____
8. On average how many patients do you see in a week (both inpatient and outpatient)? ___________
9. If you are involved in medical academics, what is your position (-s)?
   1.Instructor 2. Resident 3.Researcher 4.Others__________ 5. Not involved
10. Do you participate in any decisions on where the resources are spent/allocated like in planning and budgeting in your health care department/facility?
    1. Yes 2. No

Part II: Ethical Dilemmas

11. Below is a list of situations where medical decision-making can be difficult. **In the last two years** how often have you been in the following situations?

	Often	Some-times	Rarely	Never	Not applic-able
a) You worried if you were helping or hurting the patient with the interventions					
b) You cared for a terminally ill patient and the question on when to stop treatment or a "Do not resuscitate" order came up					
c) You were withholding (not starting) potentially life prolonging treatment to a seriously sick patient to prevent prolonging death and suffering					
d) You were withdrawing (removing) potentially life prolonging treatment to a seriously sick patient to prevent death and suffering.					
e) You felt you were over-treating patients, i.e. providing treatment or diagnostic tests they could not benefit from					
f) You were restricting treatment to a patient to give those resources to someone who could benefit more (i.e. hospital bed, ventilator, medication)					
g) You felt that the patients need of treatment was not in agreement with the patient's family needs or welfare					
h) The preferred course of treatment was not pursued because of a patient's ability to pay					
i) Limitation of resources required you to make a difficult choice					
j) Your preferred course of treatment conflicted with institutional policies, professionalcodes of ethics or laws					
k) There was significant disagreement among health care personnel on continuing treatment of the patient due to lack of resources					
l) There was significant disagreement among family members on continuing treatment of the patient					
m You were in doubt if a diagnosis should be disclosed to the patient					
n) You were in doubt to disclose sexual reproductive health problem of an adolescent under the age of consent to a parent or a guardian					
o) You were in doubt whether to reveal adult patient's medical information to the family					
p) A patient's cultural or religious views conflicted with your proposed course of treatment					
q) You were asked to help a patient to have a comfortable death or to take their own life					
r) You cared for adult patients that were not in a state to make a decision for themselves (like unconscious/ disabled), and you had to decide for them					
s) You were in doubt when asked to perform an abortion or refer a woman for abortion					
t) You were in doubt whether to provide sexual reproductive health services (i.e. contraceptives, abortion,) to someone under the age of consent					
u) You witnessed that a colleague was not acting according to professional standards(like not being honest, fair, responsible and respectful)					
v) I came across colleagues that compromise quality of care in the public system for the sake of their private practice					
w) I came across colleagues not providing appropriate care because of inadequate medical knowledge and skills					
x) I felt conflicted between my obligations to the patients in the public hospital and to my patients in private practice					

12. If you have experienced any of the situations listed in the previous table, or any other striking ethical dilemma, can you please describe a dilemma you have encountered in your own words? If you do not have enough room below, please continue on the back of the questionnaire. (If you want to respond to this question in Amharic please do so)

13. Is the situation (-s) you have described above common where you work?

1. Yes, quite common
2. Happens sometimes
3. Happens rarely
